# Non-structural carbohydrate concentrations of *Fagus sylvatica* and *Pinus sylvestris* fine roots are linked to ectomycorrhizal enzymatic activity during spring reactivation

**DOI:** 10.1007/s00572-020-00939-x

**Published:** 2020-02-20

**Authors:** Christoph Rosinger, Hans Sandén, Douglas L. Godbold

**Affiliations:** 1 Institute of Forest Ecology, University of Natural Resources and Life Sciences (BOKU), Vienna, Austria; 2 Present Address: Department of Terrestrial Ecology, Institute of Zoology, University of Cologne, Zülpicher Straße 47b, 50674 Cologne, Germany; 3 Department of Landscape Carbon Deposition, Academy of Sciences of the Czech Republic, Global Change Research Institute, Ceské Budejovice, Czech Republic

**Keywords:** Ectomycorrhizal fungi, Enzyme activity, *Fagus sylvatica*, Fine roots, Non-structural carbohydrates, *Pinus sylvestris*

## Abstract

We evaluated whether changes in fine root non-structural carbohydrate reserves of *Fagus sylvatica* and *Pinus sylvestris* trees influence potential enzymatic activities of their ectomycorrhizal symbionts from winter towards spring reactivation, and whether these changes influence potential soil enzymatic activities. We analyzed sugar and starch concentrations in the fine roots of *Fagus sylvatica* and *Pinus sylvestris* and potential activities of *ß*-glucosidase, *ß*-xylosidase, and cellobiohydrolase (as proxies for carbon-degrading enzymes) as well as leucine aminopeptidase and chitinase (as proxies for nitrogen-degrading enzymes) of their dominant ectomycorrhizal symbionts as well as in the soil. Sugar concentrations in the fine roots were significantly positively correlated with enzymatic activities of the ectomycorrhizal symbionts. In *Pinus sylvestris*, both carbon- and nitrogen-degrading enzyme activities showed significant positive correlations with fine root sugar concentrations. In *Fagus sylvatica*, fine root sugar concentrations were explicitly positively correlated with the activity of nitrogen-degrading enzymes. The chitinase activity in the soil was found to be strongly positively correlated with the enzymatic activity of the ectomycorrhizal symbionts as well as with fine root sugar concentrations. Fine root carbohydrate concentrations of *Fagus sylvatica* and *Pinus sylvestris* trees and enzymatic activities of their associated ectomycorrhizal fungi are connected. The specific nutrient demand of the tree species during spring reactivation may affect ectomycorrhizal enzymatic activity via carbon mobilization in the fine roots of *Fagus sylvatica* and *Pinus sylvestris*. Moreover, our results suggest that trees indirectly contribute to the degradation of fungal necromass by stimulating ectomycorrhizal chitinase activity in the soil.

**Electronic supplementary material:**

The online version of this article (10.1007/s00572-020-00939-x) contains supplementary material, which is available to authorized users.

## Introduction

Ectomycorrhizal (EM) fungi are pivotal drivers of biogeochemical processes in temperate and boreal forest ecosystems. Ectomycorrhizas can facilitate the majority of nitrogen (N) and phosphorus (P) uptake in exchange for photosynthetically derived carbon (C) from the host plant (Simard et al. 2003; Smith and Read 2008; van der Heijden et al. 2008). EM fungi produce a wide variety of extracellular enzymes that enables them to acquire nutrients from soil organic matter (SOM) (Pritsch and Garbaye 2011). For example, they produce peptidases and chitinases to acquire N from peptides and chitin or phosphatases to cleave organic P residues (Rineau and Courty 2011). In addition, EM fungi possess the ability to produce extracellular enzymes that depolymerize complex C compounds in order to mobilize glucose (Baber et al. 2016; Courty et al. 2007; Courty et al. 2010; Cullings et al. 2008), although this capability varies widely across lineages of EM fungi (Martin et al. 2016). This trait has been associated with potential saprotrophic capabilities of EM fungi (Bödeker et al. 2014; Cullings and Courty 2009; Hobbie et al. 2013; Lindahl and Tunlid 2015; Shah et al. 2016; Talbot et al. 2008), yet several other studies concluded that SOM decomposition by EM fungi is a by-product of the liberation of N (Averill and Hawkes 2016; Cheeke et al. 2017; Trap et al. 2017).

Tree hosts have developed mechanisms to regulate carbohydrate flows towards the fungal partner (Nehls 2008; Nehls et al. 2010) and therefore govern growth and metabolism of EM fungi (Högberg et al. 2010). A recent study by Hupperts et al. (2017) found a significant positive correlation between the amount of sugar in the fine roots and the activity of root invertase. This enzyme hydrolyzes sucrose into glucose and fructose in the interfacial apoplast. As EM fungi lack the genes encoding invertase (Smith and Read 2008), the regulation of invertase activity by the plant may dictate the amount of C allocated to EM fungi (Hupperts et al. 2017). During the growing season, the host supplies its fungal symbionts with recently fixed C (Högberg et al. 2010). However, during times of no or limited photosynthetic activity of the host, the C must either come from non-structural carbohydrate (NSC) reserves of the host (Pringle 2016), or EM fungi may obtain and metabolize C from SOM (Courty et al. 2007; Cullings and Courty 2009; Shah et al. 2016).

Undoubtedly, NSCs constitute a vast and dynamic pool of available C in trees (Körner 2003). NSC stores are mainly composed of different sugars and starch (Fischer and Höll 1991; Hoch et al. 2003) and provide C for anabolic and metabolic processes (Chapin et al. 1990). NSC reserves may also be mobilized during maximum growth in spring and early summer when the C demand exceeds C supply from photosynthesis (Hoch et al. 2003). However, most important are NSC reserves in winter and spring, where they fuel maintenance respiration (Ögren 2000), increase cold tolerance (Wong et al. 2009), and support the growth of new leaves and roots in spring (Bazot et al. 2013; Smith et al. 2017). Therefore, NSC reserves undergo seasonal variations that reflect those source-sink C dynamics. Generally, NSC reserves in temperate tree species are built up during summer when photosynthetic activity is highest and reach its peak at the end of the vegetation period (Barbaroux and Bréda 2002; Furze et al. 2018). During the dormant season, NSC pools of temperate tree species continuously deplete during the winter and were found to be lowest at bud break in spring (Dietze et al. 2014; Schädel et al. 2009).

In order to meet the plants’ specific C demand, NSC reserves can be specifically allocated to, and exchanged between, different plant organs (Bazot et al. 2013; Klein et al. 2016). For example, Smith et al. (2017) suggested that the depletion of NSC reserves in deciduous tree roots in spring was a direct consequence of increased root growth and activity during that time. As plants preferentially channel C to organs responsible for acquiring the growth-limiting resources (Johnson et al. 2013), a sufficient C supply to their mycorrhizal symbionts must have a high priority (Hartmann and Trumbore 2016). Mycorrhizal symbionts are suggested to be a strong C sink (Hartmann and Trumbore 2016; Pringle 2016), and thus, NSC concentrations and C supply to the mycorrhizal fungal partner should be connected. Loewe et al. (2000) analyzed metabolic changes and NSC dynamics of *Picea abies* seedlings during the formation of mycorrhizal symbiosis with *Amanita muscaria* and *Paxillus involutus*. They found significantly increased sucrose phosphate synthase activity and significantly decreased starch concentrations in response to mycorrhiza formation, thus suggesting that increased consumption of C by mycorrhizal symbiosis does relieve C limitation (Loewe et al. 2000). However, the relationship between NSC pools and growth as well as metabolism of EM fungi still remains poorly understood. In particular, this relationship has to our knowledge never been studied during times when supply of recently fixed C by photosynthesis is limited.

Therefore, we set out to investigate the relationship between NSC pools in the fine roots and the potential enzymatic activities of the EM fungal symbionts in a young *F. sylvatica* and a young *P. sylvestris* forest stand. We followed the two most abundant EM symbionts in each stand throughout the winter dormancy until bud break (at above-zero soil temperatures), and analyzed the potential enzymatic activities of *ß*-glucosidase, *ß*-xylosidase, and cellobiohydrolase as proxies for C-degrading enzymes, and chitinase (N-acetyl-glucosaminidase) and leucine-aminopeptidase as proxies for N-degrading enzymes (Sinsabaugh et al. 2002; Sinsabaugh et al. 2008). We hypothesized that fine root NSC reserves and ectomycorrhizal enzymatic activities are correlated during this period. In addition, we analyzed the potential activity of extracellular enzymes in the soil in order to evaluate whether or rather to which extent plant C allocation may affect the activity of extracellular enzymes in the soil.

## Methodology

### Site description

**Fig. 1:**
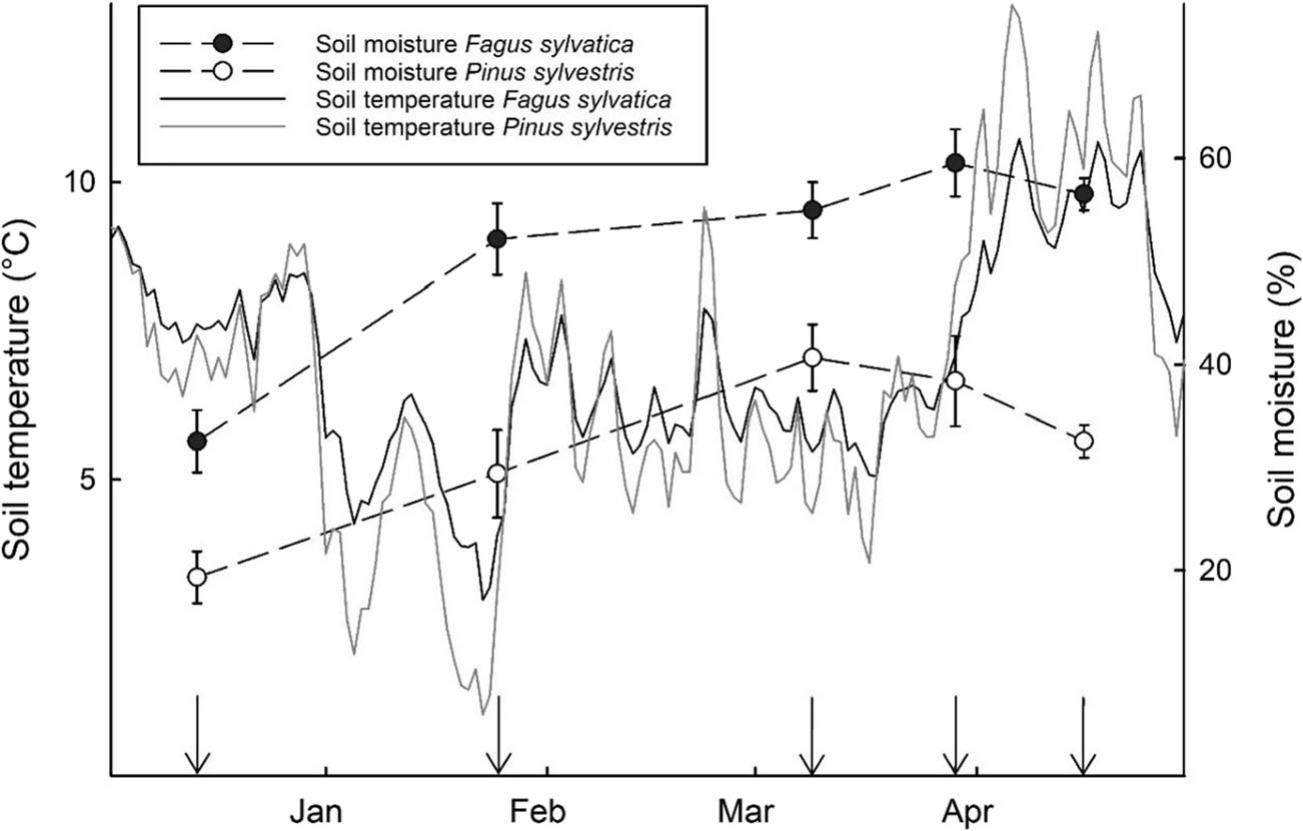
Soil temperature (solid lines) and soil moisture (dashed lines) in both *Fagus sylvatica* and *Pinus sylvestris* stands across the sampling period from December 2015 to April 2016. A mean daily temperature (*n* = 5) was calculated for both stands. Error bars of black (*F. sylvatica*) and white (*P. sylvestris*) dots denote ± 1 SE (*n* = 5). Black arrows indicate the sampling dates

The study site is a young temperate forest stand located in Central Burgenland, Austria (47° 22′ 34″ N, 16° 23′ 20″ E, 490 m a.s.l.), under the management of the Esterházy forest holding. According to Pretzsch et al. (2015), mean annual temperature and precipitation is 8.5 °C and 750 mm, respectively. The experiment was conducted on two adjacent, pure, *c*. 40 year-old *Fagus sylvatica* and *Pinus sylvestris* stands. Both plots are approximately 0.2 ha in size and South to Southwest exposed, with a slope inclination of about 35%. The bedrock consists of phyllite- and clay slates. The predominant soil types are Cambisols and Semi-Podzols (IUSS Working Group 2006). pH of the O horizon was 4.6 in the *F. sylvatica* stand and 4.4 in the *P. sylvestris* stand. Humus type is a Dys-Moder (Zanella et al. 2011). Five temperature sensors on each plot were installed to hourly record soil temperature at 5 cm depth during the experiment, and moisture content was measured gravimetrically on every sampling date (Fig. 1).

### Root/soil sampling and processing

Five sampling dates between end of leaf fall and bud break were chosen: 14.12.2015, 25.1.2016, 7.3.2016, 29.3.2016, and 16.4.2016. The last sampling date was just before bud break, when buds were already swollen and a few leaves were already visible. Within each plot, five representative sampling spots were marked. At each date, one soil core was taken within a circumference of 100 cm of each spot with a stainless steel corer (Ø 80 mm) to a depth of 10 cm. Soil cores were put in styrofoam shells and immediately transported to the laboratory, where they were kept at 3–4 °C. Soil cores were processed within 36 h of sampling. The O-horizon, with an average thickness of *c*. 2 cm in both stands was chosen for the subsequent soil and root analyses. The O-horizon was carefully separated from the soil core. First, coarse and fine roots were gently separated from the surrounding soil by shaking and the use of a brush. The soil was then passed through a 2 mm sieve and stored at 3–4 °C until further examination. The extracted roots were thoroughly rinsed under tap water and coarse roots (> 2 mm) were disregarded. The fine root (≤ 2 mm) segments were placed on wet tissue in petri dishes and stored at 3–4 °C until further examination.

### Morphotyping of ectomycorrhizas

Root segments were examined using a microscope (ZEISS Stemi 2000-CS) connected to an AxioCam ERc5s camera. First, root segments were randomly chosen and 300–400 root tips per sample were subdivided into three fractions: semivital and dead, vital non-mycorrhizal, and vital mycorrhizal root tips. The determination of different ectomycorrhizal morphotypes was based on morphological characteristics as described in Agerer (1987–2002) using macroscopic and microscopic features. Ectomycorrhizas were identified to the genus level using tip color and shape, patch color and frequency, branching pattern, mantle texture and luster, and the extent of the extramatrical hyphae (Agerer 1987–2002). As the morphotypes were not identified to species, we refer to our EM morphotypes as genus + “sp.” (Winston 1999). Matching ectomycorrhizal root tips of the two most abundant EM species were cut at a length of 2–4 mm from the root fragments using a razor blade and stored at 3–4 °C in 2 ml plastic tubes filled with tap water until further examination. After morphotyping, the remaining fine roots were immediately oven-dried for subsequent sugar and starch analyses (see below). The roots were usually dried within 48 h (in an exception case after 60 h) of the initial root extraction form the soils. During the entire processing time, the root samples were kept at 3–4 °C.

### Calculation of root tip turnover and decomposition rates

Turnover and decomposition rates of root tips were calculated using a modified decision matrix method (Brunner et al. 2013). Production was determined through increases in vital root tips, death by an increase in non-vital roots tips, and decomposition through the decrease in non-vital roots tips measure over the sampling period.

### Root tip enzyme analysis

Potential extracellular enzyme activity (from now on referred to as “EM enzyme activity”) was performed on the dissected ectomycorrhizal root tips according to the protocol of Pritsch et al. (2004) and their improvements in Pritsch et al. (2011) within 48 h of root tip dissection. Potential enzymatic activities are based on the substrates 7-amino-4-methyl coumarin (AMC; for LAP) and 4-methylumbelliferone (MUB; for all others). Substrates for enzymes as well as standard substrates were purchased from Sigma-Aldrich (MO, USA). The following enzyme activities were determined (substrate name, abbreviation, and concentration used in brackets): cellobiohydrolase (4-MUB-*ß*-D-cellobioside, CEL, 400 μM), *ß*-glucosidase (4-MUB-*ß*-D-glucopyranoside, GLU, 500 μM), leucine-aminopeptidase (L-leucine-AMC, LAP, 400 μM), N-acetyl-glucosaminidase (4-MUB-N-acetyl-*ß*-D-glucosaminide, NAG, 500 μM), and *ß*-xylosidase (4-MUB-*ß*-D-xyloside, XYL, 500 μM). Assay pH was 6.5 for LAP and 4.5 for all other substrates. Incubation times ranged between 15 and 70 min.

Previously dissected EM root tips were placed in a 96-well filter plate (AcroPrepTM filter plate, 30–40 μm mesh size, Pall Life Sciences, Crailsheim, Germany) prefilled with 150 μl of rinsing buffer. To start the assay, the rinsing buffer was vacuum-removed and discarded. Immediately after, 100 μl of the first substrate was added to the wells. The filter plate was placed on a horizontal shaker and incubated in the dark for the time of incubation. To stop the enzymatic reaction, black-well plates were prefilled with 150 μl stopping buffer and the substrate was vacuum-transferred into the black-well plate below. Standard curves containing a 50 μM AMC (for LAP) and a 50 μM MUB solution (for all other substrates) were prepared directly on the respective 96-well plates. The plate was closed with a transparent cohesive plastic film and stored in the dark at 20 °C until measurement. The filter plate was flushed with rinsing buffer and the procedure was subsequently repeated with the other substrates. Fluorescence was measured using a Perkin Elmer EnSpire multiplate reader with an excitation of 365 nm and an emission of 450 nm at 20 and 100 flashes. The potential extracellular enzyme activity was calculated using regression curves based on the standard solutions according to Pritsch et al. (2004). Immediately after the enzymatic assay, EM root tips were transferred to a transparent flat-bottom 96-well plate prefilled with 200 μl of water. To determine the surface area, root tips were scanned and analyzed using the image analysis software WinRhizo (Regent Instruments, Québec, Canada). The potential extracellular enzyme activity is expressed in nmol cm^−2^ h^−1^.

### Sugar and starch analysis

Fine root segments (≤ 2 mm) were oven-dried for 3 days at 60 °C. Immediately after drying, fine roots were ground with a ball mill at low speed and stored in air-tight plastic bags in the dark at room temperature. Measurement of soluble sugar and starch concentrations was carried out following the protocol of Chow and Landhäusser (2004). Briefly, 100 mg of root material were mixed with 5 ml of an 80% ethanol solution and incubated at 95 °C for 10 min. After centrifugation, the supernatant was removed. This extraction was repeated two more times, yielding in 15 ml ethanol containing the soluble sugars. The residues in the tube were stored wet at − 20 °C for starch analysis. Sugar concentrations were measured colorimetrically at 490 nm using phenol-sulfuric acid with a 2% phenol concentration. Sugar concentrations were determined against a glucose standard.

For the determination of starch, the residues were digested for 30 min at 50 °C using 2 ml of a 0.1 M sodium hydroxide solution. Thereafter, the solution was neutralized with 2.5 ml of a 0.1 M sodium acetate solution and 0.5 ml of an enzyme mixture containing 400 U ml^−1^ of α-amylase (ICN Biomedicals, CA, USA) and 2 U ml^−1^ of amyloglucosidase (Sigma-Aldrich) were added. This mixture was then incubated for 48 h at 50 °C. A peroxidase-glucose oxidase/*o*-dianisidine reagent (Sigma-Aldrich) was used to measure the amount of hydrolysed glucose colorimetrically at 525 nm. The obtained sugar concentrations were determined against a glucose standard using linear regression curves. The concentrations of sugar and starch are expressed as % of total dry weight.

### Soil enzyme analysis

The analysis of potential extracellular enzymatic activities in the soil (from now on referred to as “soil enzyme activity”) was performed within 4 days of sampling according to the method described in Marx et al. (2001), Saiya-Cork et al. (2002), and German et al. (2011). One gram of soil was suspended in 100 ml of a 100 mM sodium acetate buffer, pH 4.5, and homogenized for 1 min in a sonication bath. Aliquots of 200 μl were pipetted under constant stirring on a magnetic stir plate into black 96-well microplates, with four replicates for each sample. Optimal substrate concentrations and incubation times for cellobiohydrolase (CEL, 0.3 mM), *ß*-glucosidase (GLU, 0.5 mM), leucine-aminopeptidase (LAP, 1 mM), N-acetyl-glucosaminidase (NAG, 1 mM), and *ß*-xylosidase (XYL, 1 mM) were evaluated ahead (all substrates were purchased from Sigma-Aldrich). Substrates were added to each well and horizontally shaken for 30 s in order to mix the sample suspension with the substrate. The black-well plates were covered with a cohesive plastic film and incubated in the dark at 20 °C. Incubation time was 120 min. A set of standard curves for both AMC (for LAP; 50 μM, 100 μM) and MUB (for all the others; 20 μM, 50 μM, 100 μM, 250 μM) were prepared in order to cover a wide range of fluorescence. Fluorescence was measured using a Perkin Elmer EnSpire multiplate reader with an excitation of 365 nm and an emission of 450 nm at 20 and 100 flashes. The potential enzyme activity is expressed in nmol g dry soil^−1^ h^−1^.

### Statistical analyses

One-way ANOVA followed by post hoc Tukey tests were used to test whether (i) sugar and starch concentrations, (ii) potential enzymatic activities (*n* = 10–16) and ratio of C- to N-degrading enzymes of the two dominant EM symbionts, and (iii) potential soil enzymatic activities in each stand differed significantly at each of the five sampling dates (at the *p* < 0.05 level). One-way ANOVA was also used to test for significant differences between *Cenococcum* sp. associated with *Fagus sylvatica* and *Cenococcum* sp. associated with *Pinus sylvestris* (*p* < 0.05). Linear regression analyses were used to test the relationship between (i) sugar concentrations in the fine roots and EM enzymatic activity, (ii) the relationship between soil enzyme and EM enzyme activity, and (iii) the relationship between soil enzyme and the sugar concentration in the fine roots. We refer to a *p* value of < 0.05 as statistically significant. Complementary, analyses of covariance (ANCOVA) with tree species as the covariate were used to further evaluate the abovementioned relationships (*p* < 0.05). All statistical tests were performed using the software SPSS statistics 24.

## Results

### Soil temperature and moisture

Throughout the whole sampling period, the soil temperature in both stands was above 0 °C (Fig. 1). From December 1, 2015, to April 30, 2016, the mean soil temperature in 5-cm depth was 7.0 and 6.8 °C in the *Fagus sylvatica* and *Pinus sylvestris* stand, respectively. A greater fluctuation in temperature was shown in the *P. sylvestris* stand. Soil moisture in the *F. sylvatica* stand ranged from 33 to 60%, while soil moisture in the *P. sylvestris* stand ranged from 19 to 41%. Soil moisture was higher under *F. sylvatica* compared to *P. sylvestris* at all five sampling dates.

### Root properties

A total of 9579 and 7587 root tips were analyzed in the *F. sylvatica* and the *P. sylvestris* stand, respectively, during the whole sampling period. The vital fraction of the root tips including mycorrhizal and non-mycorrhizal root tips was 37 and 22% for *F. sylvatica* and *P. sylvestris*, respectively, in December 2015 (Online Resource 1). The highest share of vital root tips in the *F. sylvatica* stand was found in mid-April with 67%, while the highest share of vital root tips in the *P. sylvestris* stand was found in mid-March with 51%. Mycorrhizal root tips steadily increased from 32% in December 2015 to 67% in April 2016 in the *F. sylvatica* stand, and from 15 to 46% in the *P. sylvestris* stand. Root tip turnover was 1.09 a^−1^ and 1.80 a^−1^, and root tip decomposition was 1.35 a^−1^ and 0.97 a^−1^ for *F. sylvatica* and *P. sylvestris*, respectively.

### Sugar and starch concentrations in the fine roots

**Fig. 2:**
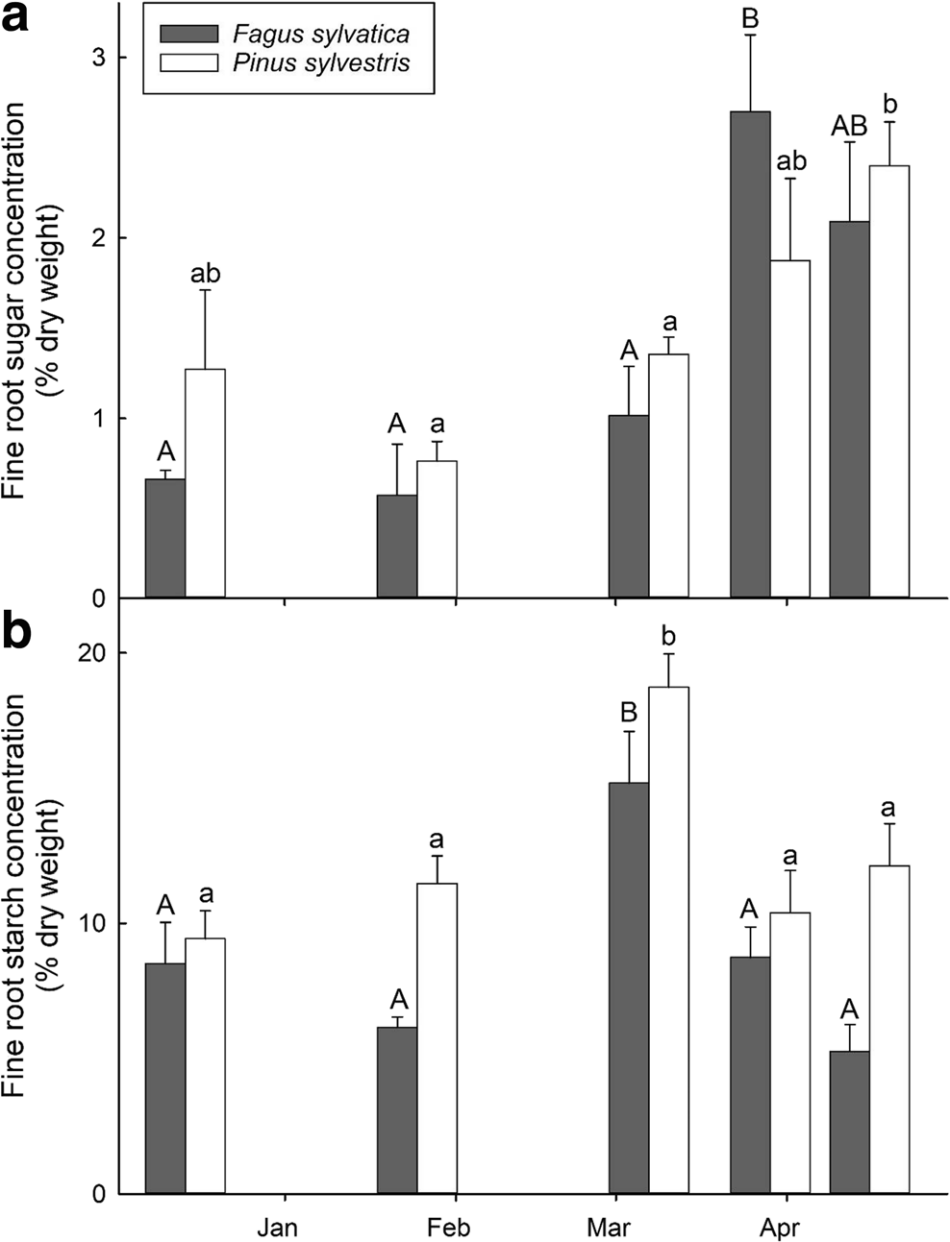
Sugar (**a**) and starch (**b**) concentrations (in % of dry weight) of *Fagus sylvatica* and *Pinus sylvestris* fine roots from December 2015 to April 2016. Error bars denote ± 1 SE (*n* = 5), and different uppercase (for *F. sylvatica*) and lowercase (for *P. sylvestris*) letters indicate significant differences at the *p* < 0.05 level

Sugar concentrations in the fine roots of *F. sylvatica* were lowest in December 2015 and January 2016. Thereafter, sugar levels increased significantly up to 2.7% (± 0.43) of total dry weight at the end of March 2016 (Fig. 2). As for the fine roots in the *P. sylvestris* stand, sugar concentrations remained low until March 2016, and thereafter significantly increased to 2.4% (± 0.24) of total dry weight in mid-April 2016.

Starch concentrations in the fine roots behaved rather similar for both tree species, i.e., a significant increase from December 2015 towards the beginning of March 2016 followed by a significant decrease towards the fourth and fifth sampling date (Fig. 2).

### Ectomycorrhizal abundance and activity

**Fig. 3:**
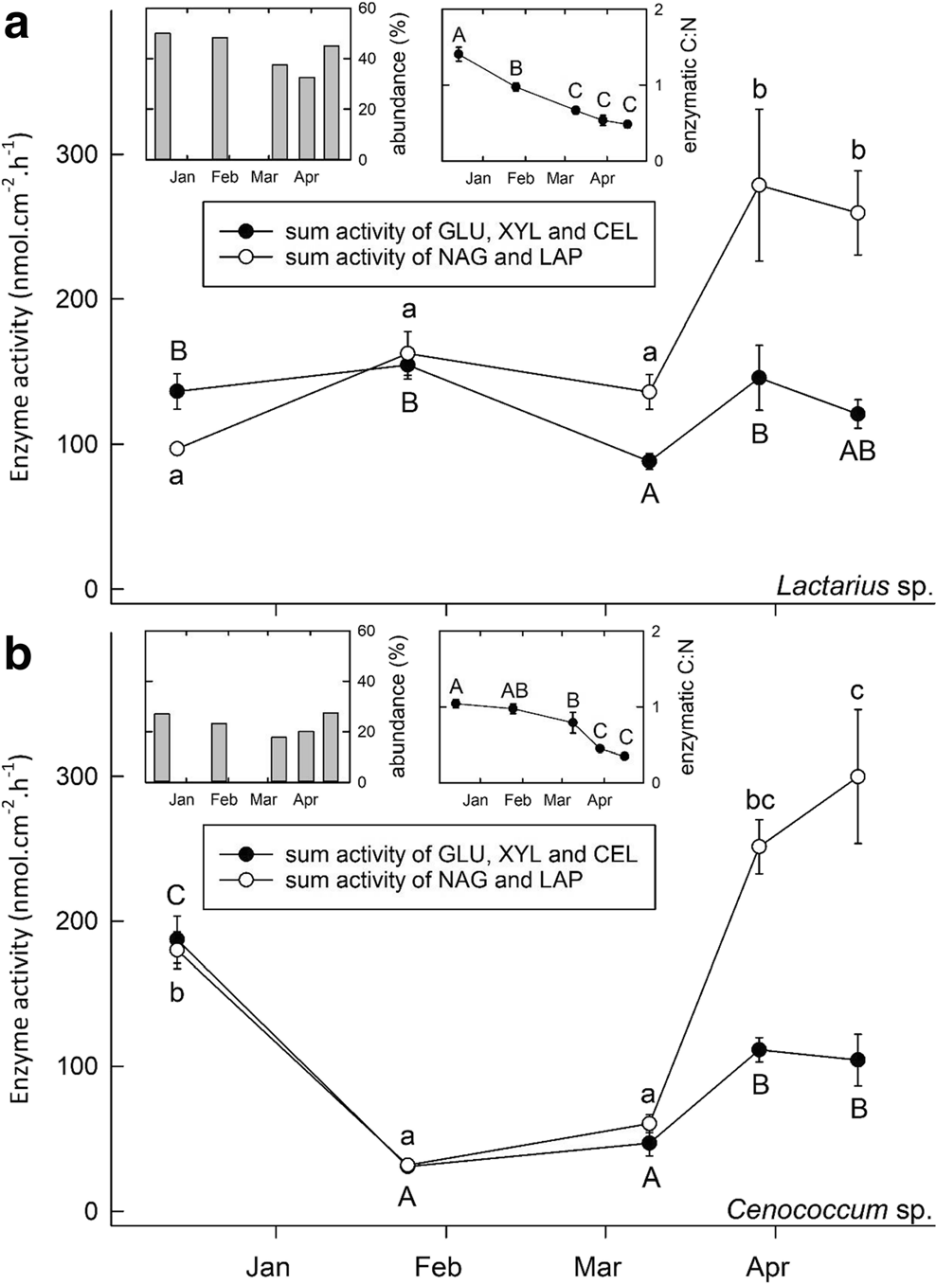
Sum activity of GLU, XYL and CEL (as C-degrading enzymes) as well as NAG and LAP (as N-degrading enzymes) (in nmol cm^−2^ h^−1^) of the two dominant EM species in the *Fagus sylvatica* stand, *Lactarius* sp. (**a**) and *Cenococcum* sp. (**b**), from December 2015 to April 2016. Error bars denote ± 1 SE (*n* = 10–16), and different uppercase (for C-degrading enzymes) and lowercase (for N-degrading enzymes) letters indicate significant differences at the *p* < 0.05 level. The chart in the upper centre displays the ratio of C- (GLU, XYL, and CEL) to N- (NAG and LAP) degrading enzymes of the respective species. Error bars denote ± 1 SE (*n* = 10–16), and different uppercase letters indicate significant differences at the *p* < 0.05 level. The chart in the upper left corner displays the abundance of the respective species within the whole EM community

In the *F. sylvatica* stand, *Lactarius* sp. and *Cenococcum* sp. were the two most dominant EM taxa. Both taxa together comprised between 55 and 77% of the total EM community (Fig. 3). As for the *P. sylvestris* stand, *Tylospora* sp. and *Cenococcum* sp. represented the two dominant EM taxa, comprising between 56 and 84% of the total EM community (Fig. 3). Pictures of the EM morphotypes can be found in the Online Resource 2.

**Fig. 4:**
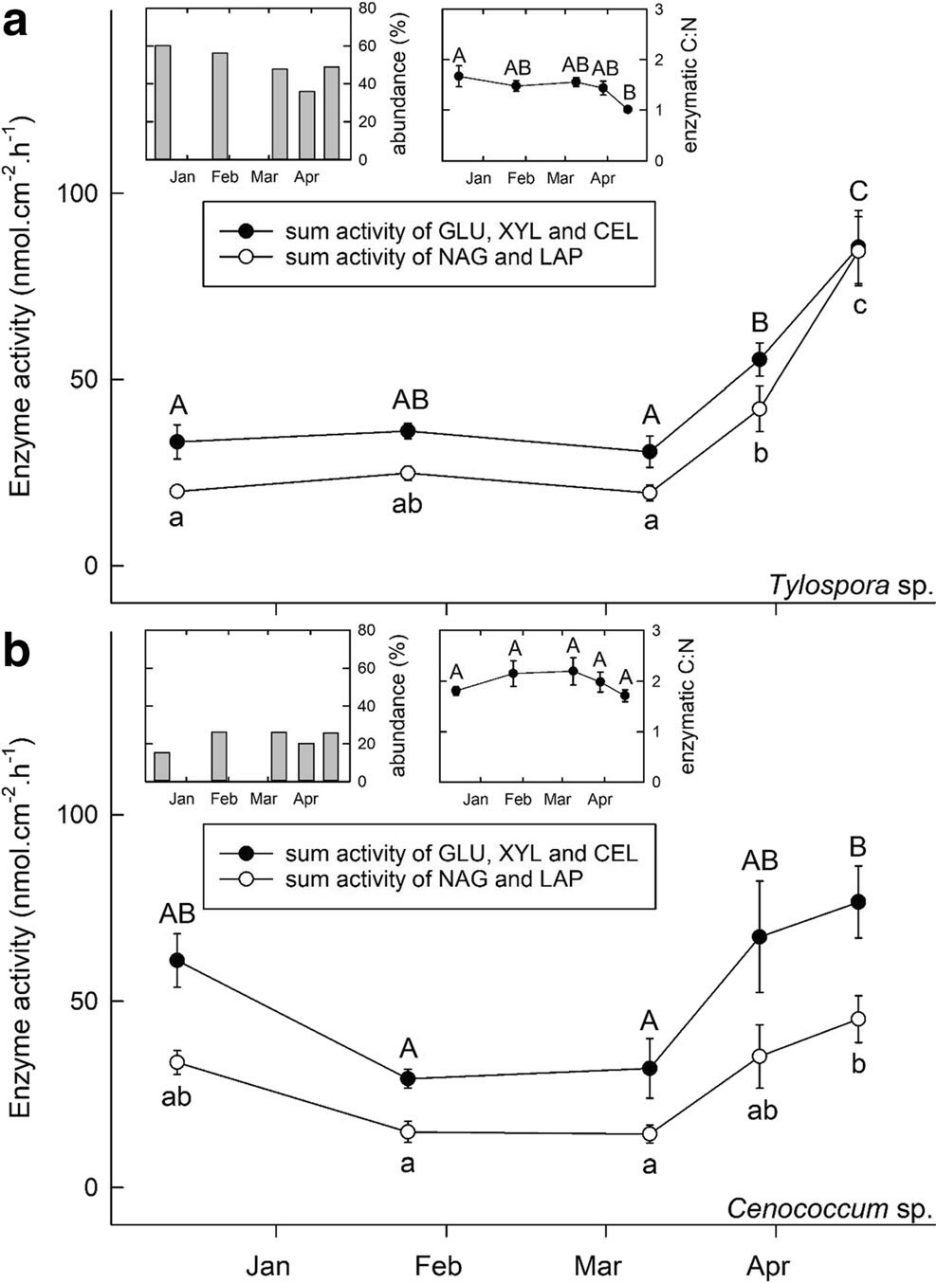
Sum activity of GLU, XYL and CEL (as C-degrading enzymes) as well as NAG and LAP (as N-degrading enzymes) (in nmol cm^−2^ h^−1^) of the two dominant EM species in the *Pinus sylvestris* stand, *Tylospora* sp. (**a**) and *Cenococcum* sp. (**b**), from December 2015 to April 2016. Error bars denote ± 1 SE (*n* = 10–16), and different uppercase (for C-degrading enzymes) and lowercase (for N-degrading enzymes) letters indicate significant differences at the *p* < 0.05 level. The chart in the upper centre displays the ratio of C- (GLU, XYL, and CEL) to N- (NAG and LAP) degrading enzymes of the respective species. Error bars denote ± 1 SE (*n* = 10–16), and different uppercase letters indicate significant differences at the *p* < 0.05 level. The chart in the upper left corner displays the abundance of the respective species within the whole EM community

Patterns were similar among GLU, XYL, and CEL (from now on referred to as “C-degrading enzymes) as well as among NAG and LAP (from now on referred to as “N-degrading enzymes”) (Tables 1 and 2). GLU activity represented around 60–70% of the total activity of C-degrading enzymes, while NAG activity represented 80–95% of the total activity of N-degrading enzymes (Tables 1 and 2). For reasons of clarity, only the sum activity of all C- and N-degrading enzymatic activities, respectively, are displayed in both Figs. 3 and 4 for the two dominant EM species. Detailed activities of all enzymes can be found in Table 1 and Table 2.

**Table 1:** Potential enzymatic activities (in nmol cm^−2^ h^−1^; LAP, leucine-aminopeptidse; NAG, N-acetyl-glucosaminidase; GLU, *ß*-glucosidase; XYL, *ß*-xylosidase; CEL, cellobiohydrolase) of the two dominant EM morphotypes (*Lactarius* sp. and *Cenococcum* sp.) in the *Fagus sylvatica* stand from December 2015 to April 2016. Given are mean values (*n* = 10–16) ± 1SE in brackets below. Different letters behind mean values indicate significant differences between sampling dates at the *p* < 0.05 level

*Fagus sylvatica* stand					
Sampling date	LAP	NAG	GLU	XYL	CEL
	(nmol cm^−2^ h^−1^)				
EM morphotype 1: *Lactarius* sp.					
14.12.2015	4.16 a	95.33 a	86.89 ab	16.20 bc	34.13 ab
	(± 0.23)	(± 4.50)	(± 7.00)	(± 1.80)	(± 2.47)
25.1.2016	9.30 ab	153.32 a	97.90 b	19.39 c	37.43 b
	(± 0.42)	(± 14.93)	(± 6.43)	(± 1.06)	(± 2.42)
9.3.2016	9.06 ab	126.89 a	57.02 a	9.44 a	21.60 a
	(± 1.30)	(± 11.12)	(± 3.64)	(± 0.70)	(± 1.57)
29.3.2016	17.49 c	265.17 b	117.54 b	16.72 bc	41.48 b
	(± 2.56)	(± 30.72)	(± 14.63)	(± 1.75)	(± 5.15)
16.4.2016	15.15 bc	245.35 b	103.02 b	11.74 ab	22.96 a
	(± 1.87)	(± 23.51)	(± 11.52)	(± 1.42)	(± 2.57)
EM morphotype 2: *Cenococcum* sp.					
14.12.2015	13.59 a	166.36 b	114.17 b	23.96 b	49.08 c
	(± 1.56)	(± 11.38)	(± 9.77)	(± 2.07)	(± 4.75)
25.1.2016	5.05 a	26.43 a	23.33 a	2.39 a	4.90 a
	(± 0.52)	(± 1.47)	(± 1.47)	(± 0.20)	(± 0.45)
9.3.2016	8.96 a	51.26 a	29.87 a	4.16 a	12.77 ab
	(± 0.76)	(± 6.15)	(± 5.50)	(± 0.51)	(± 2.88)
29.3.2016	13.61 a	238.53 bc	88.36 b	6.62 a	24.14 b
	(± 1.28)	(± 17.04)	(± 7.93)	(± 0.59)	(± 2.24)
16.4.2016	10.59 a	289.20 c	88.49 b	2.09 a	13.62 ab
	(± 4.11)	(± 47.09)	(± 14.26)	(± 0.81)	(± 3.64)

**Table 2:** Potential enzymatic activities (in nmol cm^−2^ h^−1^; LAP, leucine-aminopeptidse; NAG, N-acetyl-glucosaminidase; GLU, *ß*-glucosidase; XYL, *ß*-xylosidase; CEL, cellobiohydrolase) of the two dominant EM morphotypes (*Tylospora* sp. and *Cenococcum* sp.) in the *Pinus sylvestris* stand from December 2015 to April 2016. Given are mean values (*n* = 10–16) ± 1SE in brackets below. Different letters behind mean values indicate significant differences between sampling dates at the *p* < 0.05 level

Pinus sylvestris stand					
Sampling date	LAP	NAG	GLU	XYL	CEL
	(nmol cm^−2^ h^−1^)				
EM morphotype 1: *Tylospora* sp.					
14.12.2015	5.09 a	15.55 a	22.93 a	4.77 a	5.63 a
	(± 0.66)	(± 1.45)	(± 2.79)	(± 0.86)	(± 0.99)
25.1.2016	2.87 a	8.50 ab	12.86 a	2.81 a	3.31 a
	(± 1.28)	(± 3.80)	(± 5.75)	(± 1.26)	(± 1.48)
9.3.2016	2.82 a	16.74 a	22.75 a	2.83 a	5.04 a
	(± 1.06)	(± 2.02)	(± 3.02)	(± 0.33)	(± 1.06)
29.3.2016	3.47 a	38.67 b	39.30 a	4.49 a	11.53 ab
	(± 0.49)	(± 5.77)	(± 3.08)	(± 0.34)	(± 1.34)
16.4.2016	19.23 b	65.22 c	63.22 b	8.10 b	14.22 b
	(± 3.55)	(± 6.83)	(± 7.35)	(± 1.00)	(± 1.80)
EM morphotype 2: *Cenococcum* sp.					
14.12.2015	4.57 a	28.94 ab	45.47 ab	6.57 a	8.90 a
	(± 0.55)	(± 3.02)	(± 4.88)	(± 1.09)	(± 1.44)
25.1.2016	2.93 a	11.88 a	21.25 a	3.91 a	3.97 a
	(± 0.98)	(± 2.01)	(± 1.58)	(± 0.79)	(± 0.42)
9.3.2016	1.50 a	13.30 a	21.41 a	4.17 a	5.53 a
	(± 0.32)	(± 2.26)	(± 5.05)	(± 0.70)	(± 1.89)
29.3.2016	5.14 a	29.96 ab	46.69 ab	9.21 a	11.34 a
	(± 2.33)	(± 7.75)	(± 9.82)	(± 2.23)	(± 2.95)
16.4.2016	4.68 a	40.50 b	57.37 b	7.87 a	11.36 a
	(± 1.31)	(± 6.79)	(± 7.30)	(± 1.89)	(± 1.73)

In the *F. sylvatica* stand, the activity of N-degrading enzymes of *Lactarius* sp. was lowest in December and significantly increased towards spring reactivation in April 2016. The activity of C-degrading enzymes was highest in January 2016. In early March 2016, the activity significantly dropped, yet rose again in late March 2016 (Table 1, Fig. 3). The ratio of C- to N-degrading enzymes was highest in December 2015 and then steadily and significantly dropped towards spring reactivation. Both C- and N-degrading enzymatic activities of *Cenococcum* sp. were high in December 2015 and decreased significantly in late January 2016 and early March 2016. Thereafter, both the C- and N-degrading enzymes significantly increased towards spring reactivation. The ratio of C- to N-degrading enzymes dropped steadily and significantly towards spring reactivation (Fig. 3).

In the *P. sylvestris* stand, activity of both C- and N-degrading enzymes of *Tylospora* sp. were low from December 2015 until early March 2016 and steadily and significantly increased thereafter towards spring reactivation (Table 2, Fig. 4). The ratio of C- to N-degrading enzymes was highest in December 2015 and decreased significantly towards spring reactivation. The activity of both C- and N-degrading enzymes of *Cenococcum* sp. in the *P. sylvestris* stand was lowest in late January and early March 2016 and significantly increased in late April 2016 (Table 2, Fig. 4). The ratio of C- to N-degrading enzymes was not significantly different between all sampling dates.

Throughout the whole sampling period, *Cenococcum* sp. associated with *F. sylvatica* showed significantly higher NAG activity as compared to *Cenococcum* sp. associated with *P. sylvestris* (*p* < 0.001). LAP activity of *Cenococcum* sp. on *F. sylvatica* was significantly higher in December 2015 and on both sampling dates in early and late March 2016 (*p* < 0.01) as compared to *Cenococcum* sp. on *P. sylvestris*. GLU activity of *Cenococcum* sp. on *F. sylvatica* was significantly higher in December 2015 and at the end of March 2016 as compared to *Cenococcum* sp. on *P. sylvestris* (*p* < 0.01). XYL activity of *Cenococcum* sp. associated with *F. sylvatica* was significantly higher in December 2015, January 2016 and April 2016 as compared to *Cenococcum* sp. associated with *P. sylvestris* (*p* < 0.05), while CEL activity of *Cenococcum* sp. on *F. sylvatica* was significantly higher in December 2015 and at both sampling dates in early and late March 2016 as compared to *Cenococcum* sp. on *P. sylvestris* (*p* < 0.05).

**Fig. 5:**
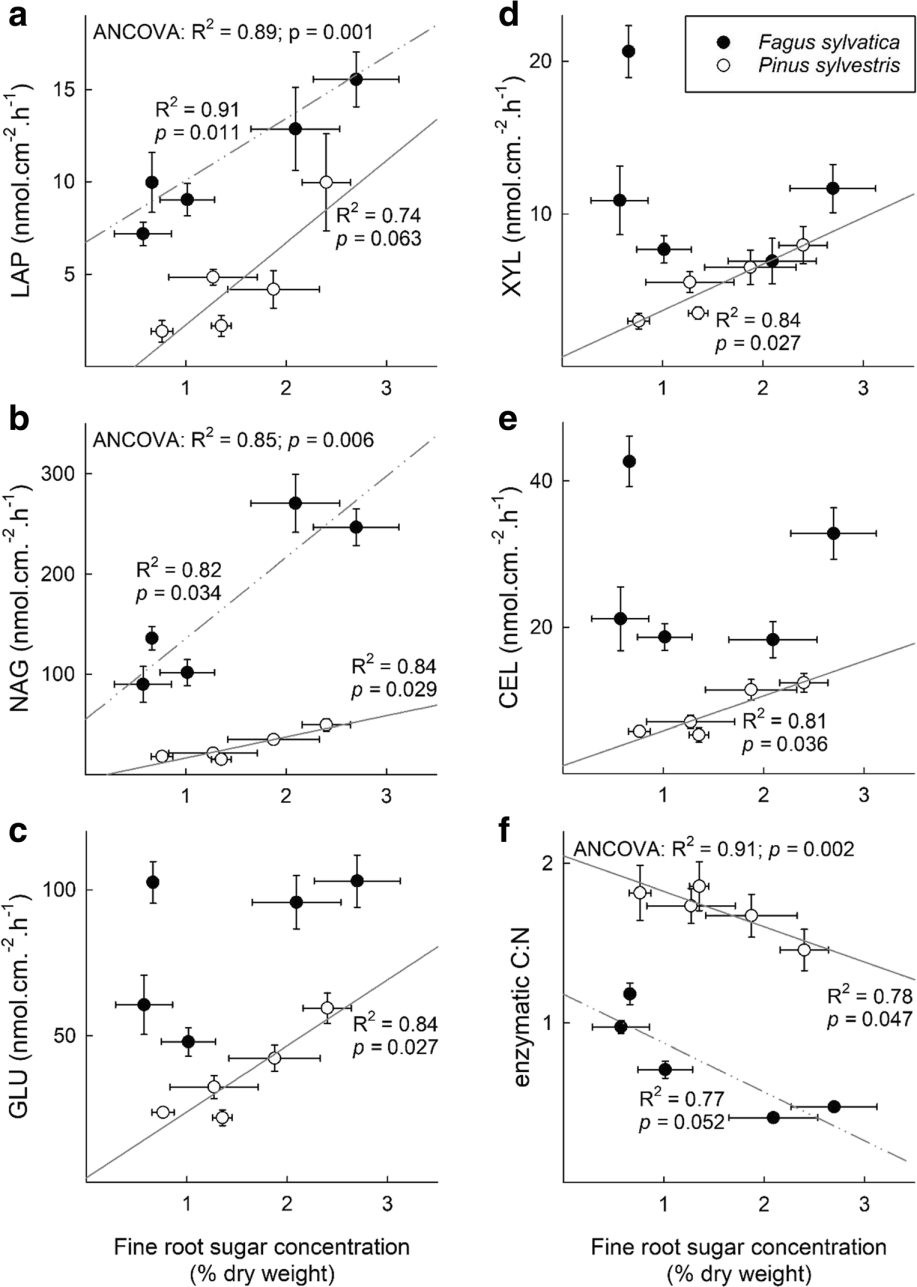
Relationship between sugar concentration in fine roots (in % dry weight) and the mean enzymatic activities (in nmol cm^−2^ h^−1^; A-E; LAP, leucine-aminopeptidase; NAG, N-acetyl-glucosaminidase; GLU, *ß*-glucosidase; XYL, *ß*-xylosidase; CEL, cellobiohydrolase) and (F) the ratio of C- (GLU, XYL and CEL) to N- (NAG and LAP) degrading enzymes of the two dominant species in a *Fagus sylvatica* (black dots) and a *Pinus sylvestris* (white dots) stand. Linear functions were used to describe the relationship between the sugar concentration and the enzymatic activities. *R*^2^ and *p* values closest to the trend lines (non-continuous, *F. sylvatica*; continuous, *P. sylvestris*) refer to the respective tree species, while *R*^2^ and *p* values in the left upper corner refer to both ecosystems as revealed by analysis of covariance (ANCOVA). R^2^ and *p* values are displayed for relationships with *p* < 0.1. Error bars denote ± 1 SE (*n*_sugar conc_ = 5; *n*_enzymes_ = 10–16)

Significantly positive relationships in the *F. sylvatica* stand were recorded between the fine root sugar concentration and the EM enzymatic activity of both NAG (*p* = 0.034) and LAP (*p* = 0.011), yet not for the activity of C-degrading enzymes nor for the ratio of C- to N-degrading enzymes (*p* = 0.052) (Fig. 5). In the *P. sylvestris* stand, significantly positive correlations were found between the sugar concentration and EM enyzmatic activities of GLU (*p* = 0.027), XYL (*p* = 0.027), CEL (*p* = 0.036), and NAG (*p* = 0.029), yet not for LAP (*p* = 0.063), and a significantly negative correlation with the ratio of C- to N-degrading enzymes (*p* = 0.047). When an analysis of covariance (ANCOVA) was applied, significant positive relationships between the sugar concentration in fine roots and the activity of LAP (*p* = 0.001) and NAG (*p* = 0.006) were detected across both tree species, yet not between GLU, XYL and CEL and fine root sugar concentrations. Moreover, a significantly negative relationship between sugar concentrations and the ratio of C- to N-degrading enzymes was found (*p* = 0.002).

### Potential soil enzymatic activity

The temporal patterns of the soil NAG and LAP activity were similar, as the concentrations increased steadily from December 2015 to April 2016 in both stands (Online Resource 3). GLU activity levels in the soil were low in December 2015 and January 2016 for both stands, and significant increased until early March 2016, after which the activity levels remained constant until April 2016. XYL activity was low in December 2015 and January 2016 in both stands, and increased significantly towards spring reactivation in April 2016. In the *P. sylvestris* stand, a significant increase in the XYL activity was already recorded in early March 2016. Activity of CEL in both stands did not show significant differences between sampling dates. No significant differences in the ratio of C- to N-degrading enzymes were found in the *F. sylvatica* and *P. sylvestris* stands across the whole sampling period.

**Fig. 6:**
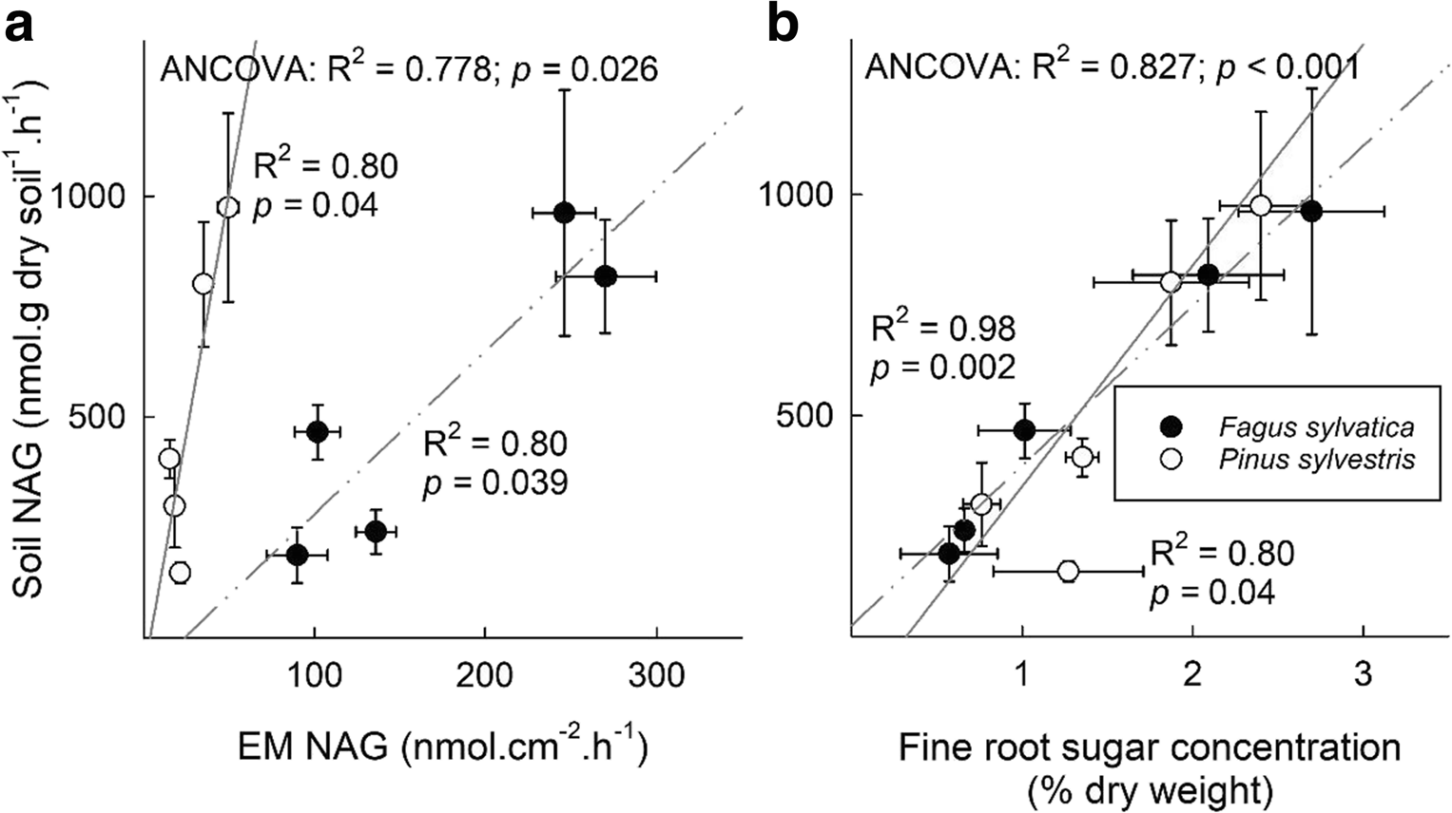
Relationship between **a** the mean NAG activities (in nmol cm^−2^ h^−1^) of the two dominant EM species as well as **b** the sugar concentrations of the fine roots (in % dry weight) and NAG activity in the soil (in nmol g dry soil^−1^ h^−1^) in a *Fagus sylvatica* (black dots) and a *Pinus sylvestris* (white dots) stand. Linear functions were used to describe the relationship between soil NAG activity and the sugar concentrations of the fine roots as well as the EM NAG activity. *R*^2^ and *p* values closest to the dots (non-continuous, *F. sylvatica*; continuous, *P. sylvestris*) refer to the respective tree species, while *R*^2^ and *p* values in the left upper corner refer to both ecosystems as revealed by analysis of covariance (ANCOVA). Error bars denote ± 1 SE (*n*_sugar conc_ = 5; *n*_soil enzymes_ = 5; *n*_root enzymes_ = 10–16)

Soil NAG activity was significantly positively correlated with the NAG activity of the two dominant EM species in both *F. sylvatica* (*p* = 0.039) and *P. sylvestris* (*p* = 0.04) stands (Fig. 6a). Such a significant relationship was not found for the other enzymes. Moreover, soil NAG activity was significantly positively correlated with the sugar concentration in the fine roots of *F. sylvatica* (*p* = 0.002) and *P. sylvestris* (*p* = 0.04) (Fig. 6b). GLU activity in the soil was also found to be significantly correlated with the sugar concentrations in the fine roots when both tree species were analyzed together using ANCOVA (*R*^*2*^ = 0.510, *p* = 0.034). No such relationships were found for LAP, XYL, and CEL.

## Discussion

### Root properties

Our study clearly shows that the transition period from winter towards spring is a period of reactivation in the soil as well as in the plants and their associated EM fungal symbionts. In general, root colonization by EM fungi is low in winter and increases towards spring, with highest colonization rates in summer (Rastin et al. 1990; Swaty et al. 1998). This pattern was found to be consistent in our study, where root colonization by EM fungi was low in winter and doubled in the *Fagus sylvatica* stand and tripled in the *Pinus sylvestris* stand towards spring reactivation. Swaty et al. (1998) suggested that the tree hosts might not be able to support their EM fungal symbionts during winter due to reduced or suppressed photosynthetic rates, thus resulting in low colonization rates during this period.

### Enzymatic activity of EM fungi

The two abundant EM taxa in the *F. sylvatica* and *P. sylvestris* stand, respectively, displayed a significant increase in both their C- and N-degrading enzymatic activity from January 2016 on towards spring reactivation (except for the C-degrading enzymatic activity of *Lactarius* sp. in the *F. sylvatica* stand; see Figs. 3 and 4), a pattern that is consistent with other studies (e.g., Buée et al. 2005; Courty et al. 2007). In general, the enzymatic activity of both EM symbionts was higher in the *F. sylvatica* stand than in the *P. sylvestris* stand. This could reflect different belowground C-use strategies of coniferous and deciduous tree species. For example, fine root biomass in *F. sylvatica* stands is higher than that in *P. sylvestris* stands in the temperate ecotone (Finér et al. 2007). Moreover, the higher root tip turnover rates of *P. sylvestris* root tips (1.80 a^−1^) as compared to *F. sylvatica* root tips (1.09 a^−1^) suggest that potentially more C is dedicated towards the production of new roots in *P. sylvestris*, while *F. sylvatica* may dedicate more C towards its fungal partner in order to explore the soil matrix and acquire nutrients.

Interestingly, *Cenococcum* sp. associated with *F. sylvatica* exhibited significantly higher enzymatic activities as compared to *Cenococcum* sp. associated with *P. sylvestris*, although both plots are in close proximity. Enzymatic activities can vary considerably within a species, and variations were often found to be linked to soil characteristics (Courty et al. 2005; Pritsch and Garbaye 2011). Species within the genera *Cenococcum* can also exhibit a high genetic variability (LoBuglio et al. 1991). For example, Jany et al. (2002) detected four different *Cenococcum geophilum* genotypes at the stand scale, and up to three genotypes appeared in one single soil core. Moreover, functional attributes can vary considerably between different genotypes (Jany et al. 2003), which could also explain the differences.

Both EM fungi in the *F. sylvatica* stand significantly increased the production of N-degrading enzymes towards spring reactivation, while the production of C-degrading enzymes did not change. As a result thereof, a significant decrease in the ratio of C- to N-degrading enzymes towards spring reactivation for *Lactarius* sp. and *Cenococcum* sp. in *F. sylvatica* was shown (Fig. 3). A minor shift in the enzymatic activity was also found for *Cenococcum* sp. in the *P. sylvestris* stand, yet not for *Tylospora* sp. (Fig. 4). Therefore, we conclude that there is a shift towards EM-derived NAG and LAP production as compared to GLU, XYL and CEL in the *F. sylvatica* stand, while resources were more equally distributed among all measured enzymes in the *P. sylvestris* stand.

### Seasonality of non-structural carbohydrate reserves

In agreement with several studies (Barbaroux and Bréda 2002; Chapin et al. 1990; Dietze et al. 2014; Ögren 2000), sugar concentrations in the fine roots of *F. sylvatica* and *P. sylvestris* showed the expected seasonal fluctuation during our sampling period, i.e., low sugar levels in winter and a fourfold increase towards spring reactivation (Fig. 2). In contrast, fine root starch concentrations of both tree species peaked in early March and thereafter decreased significantly towards spring reactivation. This clearly indicates a transfer from other reserve organs towards the fine roots (Mei et al. 2015) during early March, and a subsequent conversion to sugar (Richardson et al. 2013; Scartazza et al. 2013; Trumbore et al. 2015), potentially explaining the high fine root sugar concentrations at the succeeding sampling dates in late March and mid-April 2016.

The concentrations of sugar and starch in fine roots are well within the expected range of 2–20% (Barbaroux et al. 2003; Körner 2003) and were found to be rather similar between the fine roots of *F. sylvatica* and *P. sylvestris* (Richardson et al. 2015). According to literature, NSC reserves fluctuate stronger in deciduous trees compared to coniferous trees (Kramer and Kozlowski 1979; Piispanen and Saranpää 2001) as more C reserves must be stored and mobilized in order to support leaf flush and initial tree ring formation in spring (Hoch et al. 2003). However, NSC concentrations in fine roots do not seem to underlie such strong fluctuations.

### Linking ectomycorrhizal enzyme production and NSC reserves

We hypothesized that *Fagus sylvatica* and *Pinus sylvestris* trees influence the enzymatic activity of EM fungal taxa due to alterations in fine root NSC concentrations and subsequently C allocation during spring reactivation. Indeed, we found sugar — yet not starch — concentrations in the fine roots to be significantly positively correlated with EM-derived enzymatic activities (Fig. 5). Potential NAG and LAP activities of the dominant EM taxa were strongly correlated to sugar concentrations in the fine roots of both *F. sylvatica* and *P. sylvestris*. Those two enzymes are involved in the mobilization of N from peptides and chitin but can also be important in mobilization of C as well (Sinsabaugh and Follstad Shah 2012). NAG activity represented 80–95% of the total activity of N-degrading enzymes (Pritsch and Garbaye 2011), suggesting that the mobilization of N and potentially C from chitin, a structural polysaccharide that constitutes around 16% of the dry mass of filamentous fungi (Dahiya et al. 2006), seems particularly relevant. The mycelium of ectomycorrhizal fungi is a major contributor to the pool of microbial necromass in terrestrial forest ecosystems (Read and Perez-Moreno 2003). During winter and spring, fungal necromass has been shown to be high compared to later in the season (Ekblad et al. 2013; Wallander et al. 1997; Wallander et al. 2001), likely because tree hosts may not be able to support their EM fungal symbionts during winter due to reduced or suppressed photosynthetic rates (Swaty et al. 1998). Therefore, it seems highly plausible that plant C allocation to the EM symbiont induces the decomposition of fungal necromass via enzymatic activities during spring reactivation (Drigo et al. 2012).

In the *P. sylvestris* stand, we observed a significant correlation between sugar concentrations in fine roots and the ectomycorrhizal production of N- as well as C-degrading enzymes. In contrast, C supply to EM fungi in *F. sylvatica* seems to be particularly linked to the degradation of N-rich compounds. Different mechanisms in nutrient cycling and translocation between deciduous and coniferous tree species may account for the observed pattern (Ribbons et al. 2016). Early spring represents a period with particularly strong N demand in deciduous trees due to the establishment of new leaves. Early evidence suggests that much of the N in the leaves in deciduous trees was moved from belowground, while N translocation from the root system to the leaves in coniferous trees occurred gradually throughout the growing season (Chapin et al. 1980; Chapin and Kedrowski 1983). The establishment of new leaves during spring reactivation constitutes a major N-sink and therefore suggests a more pronounced state of N-limitation in deciduous compared to coniferous trees during this period (Marschner 2011), which could explain the observed patterns. Thus, our results substantiate that EM symbioses signify a high priority for their tree hosts, particularly during periods of increased nutrient requirement (Högberg et al. 2003; Zhang et al. 2015).

### Relationship between EM enzymatic activities and soil enzymatic activities

Higher plant photosynthetic activity during spring reactivation not only promotes EM fungal activity, but soil microbial activity in general (Kaiser et al. 2010; Žifčáková et al. 2016). All potential C- and N-degrading enzymatic activities except of CEL increased in both forests towards spring reactivation (Rastin et al. 1988). While activities of NAG and LAP increased rather synchronously with sugar concentrations in the fine roots, GLU activities in both forest stands were already high at the beginning of March, suggesting that the activity of this enzyme is a trait of microbial groups other than ectomycorrhizal fungi and rhizosphere bacteria (Kaiser et al. 2010).

The relative and absolute contribution of EM fungi and other microbial groups to enzyme production in soil remains rather ambiguous (Phillips et al. 2014; Talbot et al. 2013). In our study, we found a strong relationship between EM-derived NAG activity and soil NAG activity (Fig. 6a), yet neither LAP nor the C-degrading enzymes showed this relationship. This suggests that EM fungi contribute substantially to the degradation of chitin during spring reactivation and supports earlier studies suggesting that NAG activity is an indicator for fungal activity in general (Hodge et al. 1995; Miller et al. 1998). Moreover, sugar concentrations in the fine roots and NAG activity in the soil were significantly positively correlated in both forest stands (Fig. 6b), revealing a direct and significant link between the host’s C status in the fine roots and the enzymatic activity of microbes in the soil.

## Conclusion

In this study, we set out to investigate the relationship between fine root NSC reserves and the enzymatic activity of EM fungal symbionts in a *Fagus sylvatica* and a *Pinus sylvestris* stand. We hypothesized that non-structural carbohydrate reserves in the fine roots and the production of C- and N-degrading enzymes of the EM fungal symbionts are related during spring reactivation. To our knowledge, we show for the first time that sugar concentrations in the fine roots are significantly positively correlated with EM-derived enzymatic activities. In the *P. sylvestris* stand, both C- and N-degrading enzymes showed significant positive correlations with fine root sugar concentrations. In the *F. sylvatica* stand, sugar concentrations in the fine roots were explicitly correlated with the activity of N-degrading enzymes of the EM fungal symbionts. This may reflect differences in the specific N demand during spring reactivation between both tree species. While the period of spring reactivation exerts a strong N demand in deciduous trees due to the establishment of new leaves, N demand in coniferous trees is rather equally strong throughout the growing season. Therefore, our analysis reinforces the view of a host-dependent regulation of the activity of EM fungi (Nehls et al. 2001). Moreover, EM fungi contribute substantially to the activity of NAG in the soil during spring reactivation. We suggest that fungal necromass is broken down by EM fungi to mobilize N, and potentially C, from chitin (Drigo et al. 2012). Thus, the trees´ resource demand may directly affect the mineralization of SOM.

## Electronic supplementary material

ESM 1 (DOCX 2862 kb)

## Abbreviations

C: Carbon
CEL: Cellobiohydroloase
EM: Ectomycorrhizal
GLU: *ß*-Glucosidase
LAP: Leucine-aminopeptidase
N: Nitrogen
NAG: N-acetyl-glucosaminidase
NSC: Non-structural carbohydrates
P: Phosphorus
SOM: Soil organic matter
XYL: *ß*-Xylosidase

## References

[CR1] Agerer R (1987). Colour atlas of ectomycorrhizae.

[CR2] Averill C, Hawkes CV (2016). Ectomycorrhizal fungi slow soil carbon cycling. Ecol Lett.

[CR3] Baber K, Otto P, Kahl T, Gossner MM, Wirth C, Gminder A, Bässler C (2016). Disentangling the effects of forest-stand type and dead-wood origin of the early successional stage on the diversity of wood-inhabiting fungi. For Ecol Manag.

[CR4] Barbaroux C, Bréda N (2002). Contrasting distribution and seasonal dynamics of carbohydrate reserves in stem wood of adult ring-porous sessile oak and diffuse-porous beech trees. Tree Physiol.

[CR5] Barbaroux C, Bréda N, Dufrêne E (2003). Distribution of above-ground and below-ground carbohydrate reserves in adult trees of two contrasting broad-leaved species (Quercus petraea and Fagus sylvatica). New Phytol.

[CR6] Bazot S, Barthes L, Blanot D, Fresneau C (2013). Distribution of non-structural nitrogen and carbohydrate compounds in mature oak trees in a temperate forest at four key phenological stages. Trees.

[CR7] Bödeker I, Clemmensen KE, Boer W, Martin F, Olson Å, Lindahl BD (2014). Ectomycorrhizal Cortinarius species participate in enzymatic oxidation of humus in northern forest ecosystems. New Phytol.

[CR8] Brunner I, Bakker MR, Björk RG, Hirano Y, Lukac M, Aranda X, Børja I, Eldhuset TD, Helmisaari H-S, Jourdan C (2013). Fine-root turnover rates of European forests revisited: an analysis of data from sequential coring and ingrowth cores. Plant Soil.

[CR9] Buée M, Vairelles D, Garbaye J (2005). Year-round monitoring of diversity and potential metabolic activity of the ectomycorrhizal community in a beech (Fagus silvatica) forest subjected to two thinning regimes. Mycorrhiza.

[CR10] Chapin FS, Johnson DA, McKendrick JD (1980) Seasonal movement of nutrients in plants of differing growth form in an Alaskan tundra ecosystem: implications for herbivory. J Ecol:189–209

[CR11] Chapin FS, Kedrowski RA (1983). Seasonal changes in nitrogen and phosphorus fractions and autumn retranslocation in evergreen and deciduous taiga trees. Ecology.

[CR12] Chapin FS, Schulze E, Mooney HA (1990). The ecology and economics of storage in plants. Annu Rev Ecol Syst.

[CR13] Cheeke TE, Phillips RP, Brzostek ER, Rosling A, Bever JD, Fransson P (2017). Dominant mycorrhizal association of trees alters carbon and nutrient cycling by selecting for microbial groups with distinct enzyme function. New Phytol.

[CR14] Chow PS, Landhäusser SM (2004). A method for routine measurements of total sugar and starch content in woody plant tissues. Tree Physiol.

[CR15] Courty P-E, Bréda N, Garbaye J (2007). Relation between oak tree phenology and the secretion of organic matter degrading enzymes by Lactarius quietus ectomycorrhizas before and during bud break. Soil Biol Biochem.

[CR16] Courty P-E, Franc A, Garbaye J (2010). Temporal and functional pattern of secreted enzyme activities in an ectomycorrhizal community. Soil Biol Biochem.

[CR17] Courty PE, Pritsch K, Schloter M, Hartmann A, Garbaye J (2005). Activity profiling of ectomycorrhiza communities in two forest soils using multiple enzymatic tests. New Phytol.

[CR18] Cullings K, Courty P-E (2009). Saprotrophic capabilities as functional traits to study functional diversity and resilience of ectomycorrhizal community. Oecologia.

[CR19] Cullings K, Ishkhanova G, Henson J (2008). Defoliation effects on enzyme activities of the ectomycorrhizal fungus Suillus granulatus in a Pinus contorta (lodgepole pine) stand in Yellowstone National Park. Oecologia.

[CR20] Dahiya N, Tewari R, Hoondal GS (2006). Biotechnological aspects of chitinolytic enzymes: a review. Appl Microbiol Biotechnol.

[CR21] Dietze MC, Sala A, Carbone MS, Czimczik CI, Mantooth JA, Richardson AD, Vargas R (2014). Nonstructural carbon in woody plants. Annu Rev Plant Biol.

[CR22] Drigo B, Anderson IC, Kannangara G, Cairney JW, Johnson D (2012). Rapid incorporation of carbon from ectomycorrhizal mycelial necromass into soil fungal communities. Soil Biol Biochem.

[CR23] Ekblad A, Wallander H, Godbold D, Cruz C, Johnson D, Baldrian P, Björk R, Epron D, Kieliszewska-Rokicka B, Kjøller R (2013). The production and turnover of extramatrical mycelium of ectomycorrhizal fungi in forest soils: role in carbon cycling. Plant Soil.

[CR24] Finér L, Helmisaari H-S, Lõhmus K, Majdi H, Brunner I, Børja I, Eldhuset T, Godbold D, Grebenc T, Konôpka B (2007). Variation in fine root biomass of three European tree species: beech (Fagus sylvatica L.), Norway spruce (Picea abies L. Karst.), and scots pine (Pinus sylvestris L.). Plant Biosyst.

[CR25] Fischer C, Höll W (1991). Food reserves of Scots pine (Pinus sylvestris L.). Trees.

[CR26] Furze ME, Huggett BA, Aubrecht DM, Stolz CD, Carbone MS, Richardson AD (2018). Whole-tree nonstructural carbohydrate storage and seasonal dynamics in five temperate species. New Phytol.

[CR27] German DP, Weintraub MN, Grandy AS, Lauber CL, Rinkes ZL, Allison SD (2011). Optimization of hydrolytic and oxidative enzyme methods for ecosystem studies. Soil Biol Biochem.

[CR28] Hartmann H, Trumbore S (2016). Understanding the roles of nonstructural carbohydrates in forest trees–from what we can measure to what we want to know. New Phytol.

[CR29] Hobbie EA, Ouimette AP, Schuur EA, Kierstead D, Trappe JM, Bendiksen K, Ohenoja E (2013). Radiocarbon evidence for the mining of organic nitrogen from soil by mycorrhizal fungi. Biogeochemistry.

[CR30] Hoch G, Richter A, Körner C (2003). Non-structural carbon compounds in temperate forest trees. Plant Cell Environ.

[CR31] Hodge A, Alexander IJ, Gooday GW (1995). Chitinolytic enzymes of pathogenic and ectomycorrhizal fungi. Mycol Res.

[CR32] Högberg MN, Bååth E, Nordgren A, Arnebrant K, Högberg P (2003). Contrasting effects of nitrogen availability on plant carbon supply to mycorrhizal fungi and saprotrophs–a hypothesis based on field observations in boreal forest. New Phytol.

[CR33] Högberg MN, Briones MJ, Keel SG, Metcalfe DB, Campbell C, Midwood AJ, Thornton B, Hurry V, Linder S, Näsholm T (2010). Quantification of effects of season and nitrogen supply on tree below-ground carbon transfer to ectomycorrhizal fungi and other soil organisms in a boreal pine forest. New Phytol.

[CR34] Hupperts SF, Karst J, Pritsch K, Landhäusser SM (2017). Host phenology and potential saprotrophism of ectomycorrhizal fungi in the boreal forest. Funct Ecol.

[CR35] IUSS Working Group W (2006) World reference base for soil resources. World Soil Resour Rep 103

[CR36] Jany J-L, Martin F, Garbaye J (2003). Respiration activity of ectomycorrhizas from Cenococcum geophilum and Lactarius sp. in relation to soil water potential in five beech forests. Plant Soil.

[CR37] Jany JL, Garbaye J, Martin F (2002). Cenococcum geophilum populations show a high degree of genetic diversity in beech forests. New Phytol.

[CR38] Johnson NC, Angelard C, Sanders IR, Kiers ET (2013). Predicting community and ecosystem outcomes of mycorrhizal responses to global change. Ecol Lett.

[CR39] Kaiser C, Koranda M, Kitzler B, Fuchslueger L, Schnecker J, Schweiger P, Rasche F, Zechmeister-Boltenstern S, Sessitsch A, Richter A (2010). Belowground carbon allocation by trees drives seasonal patterns of extracellular enzyme activities by altering microbial community composition in a beech forest soil. New Phytol.

[CR40] Klein T, Vitasse Y, Hoch G (2016). Coordination between growth, phenology and carbon storage in three coexisting deciduous tree species in a temperate forest. Tree Physiol.

[CR41] Körner C (2003). Carbon limitation in trees. J Ecol.

[CR42] Kramer P, Kozlowski T (1979). Physiology of woody plants.

[CR43] Lindahl BD, Tunlid A (2015). Ectomycorrhizal fungi–potential organic matter decomposers, yet not saprotrophs. New Phytol.

[CR44] LoBuglio KF, Rogers SO, Wang C (1991). Variation in ribosomal DNA among isolates of the mycorrhizal fungus Cenococcum geophilum. Can J Bot.

[CR45] Loewe A, Einig W, Shi L, Dizengremel P, Hampp R (2000). Mycorrhiza formation and elevated CO 2 both increase the capacity for sucrose synthesis in source leaves of spruce and aspen. New Phytol.

[CR46] Marschner H (2011) Marschner's mineral nutrition of higher plants. Academic press

[CR47] Martin F, Kohler A, Murat C, Veneault-Fourrey C, Hibbett DS (2016). Unearthing the roots of ectomycorrhizal symbioses. Nat Rev Microbiol.

[CR48] Marx M-C, Wood M, Jarvis S (2001). A microplate fluorimetric assay for the study of enzyme diversity in soils. Soil Biol Biochem.

[CR49] Mei L, Xiong Y, Gu J, Wang Z, Guo D (2015). Whole-tree dynamics of non-structural carbohydrate and nitrogen pools across different seasons and in response to girdling in two temperate trees. Oecologia.

[CR50] Miller M, Palojärvi A, Rangger A, Reeslev M, Kjøller A (1998). The use of fluorogenic substrates to measure fungal presence and activity in soil. Appl Environ Microbiol.

[CR51] Nehls U (2008). Mastering ectomycorrhizal symbiosis: the impact of carbohydrates. J Exp Bot.

[CR52] Nehls U, Göhringer F, Wittulsky S, Dietz S (2010). Fungal carbohydrate support in the ectomycorrhizal symbiosis: a review. Plant Biol.

[CR53] Nehls U, Mikolajewski S, Magel E, Hampp R (2001). Carbohydrate metabolism in ectomycorrhizas: gene expression, monosaccharide transport and metabolic control. New Phytol.

[CR54] Ögren E (2000). Maintenance respiration correlates with sugar but not nitrogen concentration in dormant plants. Physiol Plant.

[CR55] Phillips LA, Ward V, Jones MD (2014). Ectomycorrhizal fungi contribute to soil organic matter cycling in sub-boreal forests. ISME J.

[CR56] Piispanen R, Saranpää P (2001). Variation of non-structural carbohydrates in silver birch (Betula pendula Roth) wood. Trees.

[CR57] Pretzsch H, Del Río M, Ammer C, Avdagic A, Barbeito I, Bielak K, Brazaitis G, Coll L, Dirnberger G, Drössler L (2015). Growth and yield of mixed versus pure stands of scots pine (Pinus sylvestris L.) and European beech (Fagus sylvatica L.) analysed along a productivity gradient through Europe. Eur J For Res.

[CR58] Pringle EG (2016). Integrating plant carbon dynamics with mutualism ecology. New Phytol.

[CR59] Pritsch K, Courty PE, Churin J-L, Cloutier-Hurteau B, Ali MA, Damon C, Duchemin M, Egli S, Ernst J, Fraissinet-Tachet L (2011). Optimized assay and storage conditions for enzyme activity profiling of ectomycorrhizae. Mycorrhiza.

[CR60] Pritsch K, Garbaye J (2011). Enzyme secretion by ECM fungi and exploitation of mineral nutrients from soil organic matter. Ann For Sci.

[CR61] Pritsch K, Raidl S, Marksteiner E, Blaschke H, Agerer R, Schloter M, Hartmann A (2004). A rapid and highly sensitive method for measuring enzyme activities in single mycorrhizal tips using 4-methylumbelliferone-labelled fluorogenic substrates in a microplate system. J Microbiol Methods.

[CR62] Rastin N, Rosenplänter K, Hüttermann A (1988). Seasonal variation of enzyme activity and their dependence on certain soil factors in a beech forest soil. Soil Biol Biochem.

[CR63] Rastin N, Schlechte G, Hüttermann A, Rosenplänter K (1990). Seasonal fluctuation of some biological and biochemical soil factors and their dependence on certain soil factors on the upper and lower slope of a spruce forest. Soil Biol Biochem.

[CR64] Read D, Perez-Moreno J (2003). Mycorrhizas and nutrient cycling in ecosystems–a journey towards relevance?. New Phytol.

[CR65] Ribbons RR, Levy-Booth DJ, Masse J, Grayston SJ, McDonald MA, Vesterdal L, Prescott CE (2016). Linking microbial communities, functional genes and nitrogen-cycling processes in forest floors under four tree species. Soil Biol Biochem.

[CR66] Richardson AD, Carbone MS, Huggett BA, Furze ME, Czimczik CI, Walker JC, Xu X, Schaberg PG, Murakami P (2015). Distribution and mixing of old and new nonstructural carbon in two temperate trees. New Phytol.

[CR67] Richardson AD, Carbone MS, Keenan TF, Czimczik CI, Hollinger DY, Murakami P, Schaberg PG, Xu X (2013). Seasonal dynamics and age of stemwood nonstructural carbohydrates in temperate forest trees. New Phytol.

[CR68] Rineau F, Courty P-E (2011). Secreted enzymatic activities of ectomycorrhizal fungi as a case study of functional diversity and functional redundancy. Ann For Sci.

[CR69] Saiya-Cork K, Sinsabaugh R, Zak D (2002). The effects of long term nitrogen deposition on extracellular enzyme activity in an Acer saccharum forest soil. Soil Biol Biochem.

[CR70] Scartazza A, Moscatello S, Matteucci G, Battistelli A, Brugnoli E (2013). Seasonal and inter-annual dynamics of growth, non-structural carbohydrates and C stable isotopes in a Mediterranean beech forest. Tree Physiol.

[CR71] Schädel C, Blöchl A, Richter A, Hoch G (2009). Short-term dynamics of nonstructural carbohydrates and hemicelluloses in young branches of temperate forest trees during bud break. Tree Physiol.

[CR72] Shah F, Nicolás C, Bentzer J, Ellström M, Smits M, Rineau F, Canbäck B, Floudas D, Carleer R, Lackner G (2016). Ectomycorrhizal fungi decompose soil organic matter using oxidative mechanisms adapted from saprotrophic ancestors. New Phytol.

[CR73] Simard SW, Jones MD, Durall DM (2003) Carbon and nutrient fluxes within and between mycorrhizal plants. Mycorrhizal ecology. Springer

[CR74] Sinsabaugh RL, Carreiro MM, Alvarez S (2002). Enzyme and microbial dynamics of litter decomposition.

[CR75] Sinsabaugh RL, Follstad Shah JJ (2012). Ecoenzymatic stoichiometry and ecological theory. Annu Rev Ecol Evol Syst.

[CR76] Sinsabaugh RL, Lauber CL, Weintraub MN, Ahmed B, Allison SD, Crenshaw C, Contosta AR, Cusack D, Frey S, Gallo ME (2008). Stoichiometry of soil enzyme activity at global scale. Ecol Lett.

[CR77] Smith MG, Miller RE, Arndt SK, Kasel S, Bennett LT (2017). Whole-tree distribution and temporal variation of non-structural carbohydrates in broadleaf evergreen trees. Tree Physiol.

[CR78] Smith S, Read D (2008). Mycorrhizal symbiosis.

[CR79] Swaty RL, Gehring CA, Van Ert M, Theimer TC, Keim P, Whitham TG (1998). Temporal variation in temperature and rainfall differentially affects ectomycorrhizal colonization at two contrasting sites. New Phytol.

[CR80] Talbot J, Allison S, Treseder K (2008). Decomposers in disguise: mycorrhizal fungi as regulators of soil C dynamics in ecosystems under global change. Funct Ecol.

[CR81] Talbot JM, Bruns TD, Smith DP, Branco S, Glassman SI, Erlandson S, Vilgalys R, Peay KG (2013). Independent roles of ectomycorrhizal and saprotrophic communities in soil organic matter decomposition. Soil Biol Biochem.

[CR82] Trap J, Akpa-Vinceslas M, Margerie P, Boudsocq S, Richard F, Decaëns T, Aubert M (2017). Slow decomposition of leaf litter from mature Fagus sylvatica trees promotes offspring nitrogen acquisition by interacting with ectomycorrhizal fungi. J Ecol.

[CR83] Trumbore S, Czimczik CI, Sierra CA, Muhr J, Xu X (2015). Non-structural carbon dynamics and allocation relate to growth rate and leaf habit in California oaks. Tree Physiol.

[CR84] van der Heijden MG, Bardgett RD, van Straalen NM (2008). The unseen majority: soil microbes as drivers of plant diversity and productivity in terrestrial ecosystems. Ecol Lett.

[CR85] Wallander H, Massicotte H, Nylund J (1997). Seasonal variation in ergosterol, chitin and protein in ectomycorrhizal roots collected in a Swedish pine forest. Soil Biol Biochem.

[CR86] Wallander H, Nilsson LO, Hagerberg D, Bååth E (2001). Estimation of the biomass and seasonal growth of external mycelium of ectomycorrhizal fungi in the field. New Phytol.

[CR87] Winston JE (1999) Describing species: practical taxonomic procedure for biologists. Columbia University Press

[CR88] Wong B, Baggett K, Rye A (2009). Cold-season patterns of reserve and soluble carbohydrates in sugar maple and ice-damaged trees of two age classes following drought. Botany.

[CR89] Zanella A, Jabiol B, Ponge J-F, Sartori G, De Waal R, Van Delft B, Graefe U, Cools N, Katzensteiner K, Hager H (2011). A European morpho-functional classification of humus forms. Geoderma.

[CR90] Zhang H, Ziegler W, Han X, Trumbore S, Hartmann H (2015). Plant carbon limitation does not reduce nitrogen transfer from arbuscular mycorrhizal fungi to Plantago lanceolata. Plant Soil.

[CR91] Žifčáková L, Větrovský T, Howe A, Baldrian P (2016). Microbial activity in forest soil reflects the changes in ecosystem properties between summer and winter. Environ Microbiol.

