# Supplementary material for: Non-structural carbohydrate concentrations of Fagus sylvatica and Pinus sylvestris fine roots are linked to ectomycorrhizal enzymatic activity during spring reactivation
Source: Mycorrhiza. 2020 Feb 20;30(2):197–210. doi: 10.1007/s00572-020-00939-x (PMC7228962; doi:10.1007/s00572-020-00939-x)
Supplement: Supplementary file 1 — (DOCX 2862 kb) [file 572_2020_939_MOESM1_ESM.docx]

**Online Resources**

**Title:** Non-structural carbohydrate concentrations of *Fagus sylvatica* and *Pinus sylvestris* fine roots are linked to ectomycorrhizal enzymatic activity during spring reactivation

**Journal Name:** Mycorrhiza

**Authors:** Christoph Rosinger, Hans Sandén, Douglas L. Godbold

**Corresponding author:** Christoph Rosinger; Institute of Forest Ecology, University of Natural Resources and Life Sciences (BOKU), Vienna, Austria; christoph.rosinger@boku.ac.at

**Online Resource 1** Fractions of non-mycorrhizal, mycorrhizal and semi-vital/broken/dead root tips (in %) in the Fagus sylvatica and Pinus sylvestris stand from December 2015 to April 2016

|  | **Fagus sylvatica** | | | |  |
| --- | --- | --- | --- | --- | --- |
|  | non-mycorrhizal | mycorrhizal | semi-vital/broken/ dead | |  |
| ***Sampling date*** | (%) | | | |  |
| *14.12.2015* | 4.70 | 31.94 | 63.36 | |  |
| *25.1.2016* | 3.89 | 38.99 | 57.12 | |  |
| *9.3.2016* | 20.45 | 39.69 | 39.86 | |  |
| *29.3.2016* | 0.12 | 51.72 | 48.16 | |  |
| *16.4.2016* | 0.66 | 67.00 | 32.34 | |  |
|  |  |  |  | |  |
|  | **Pinus sylvestris** | | | |  |
|  | non-mycorrhizal | mycorrhizal | | semi-vital/broken/ dead | |
| ***Sampling date*** | (%) | | | |  |
| *14.12.2015* | 7.00 | 14.67 | 77.99 | |  |
| *25.1.2016* | 3.97 | 21.50 | 74.53 | |  |
| *9.3.2016* | 4.54 | 46.20 | 49.27 | |  |
| *29.3.2016* | 2.13 | 43.15 | 54.72 | |  |
| *16.4.2016* | 0.71 | 45.61 | 53.68 | |  |
|  |  |  |  | |  |


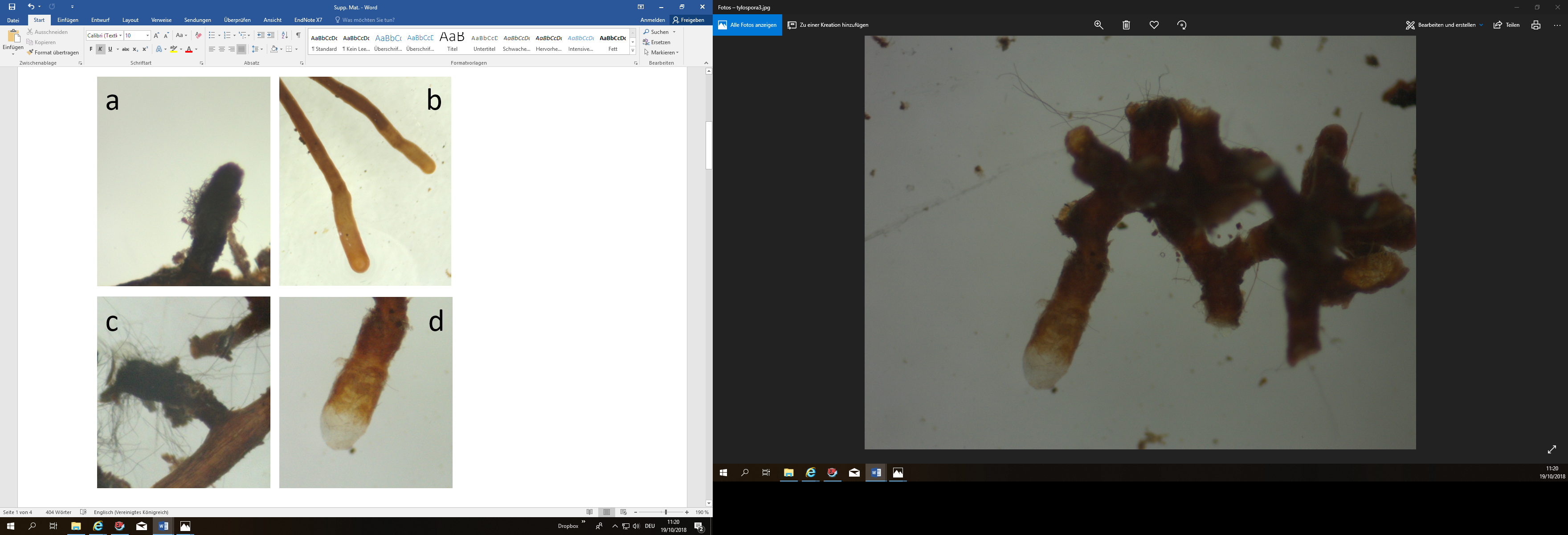


**Online Resource 2** Pictures of the dominant EM morphotypes in the Fagus sylvatica (a,b) and the Pinus sylvestris (c,d) stand; (a) Cenococcum sp.; (b) Lactarius sp.; (c) Cenococcum sp.; (d) Tylospora sp.


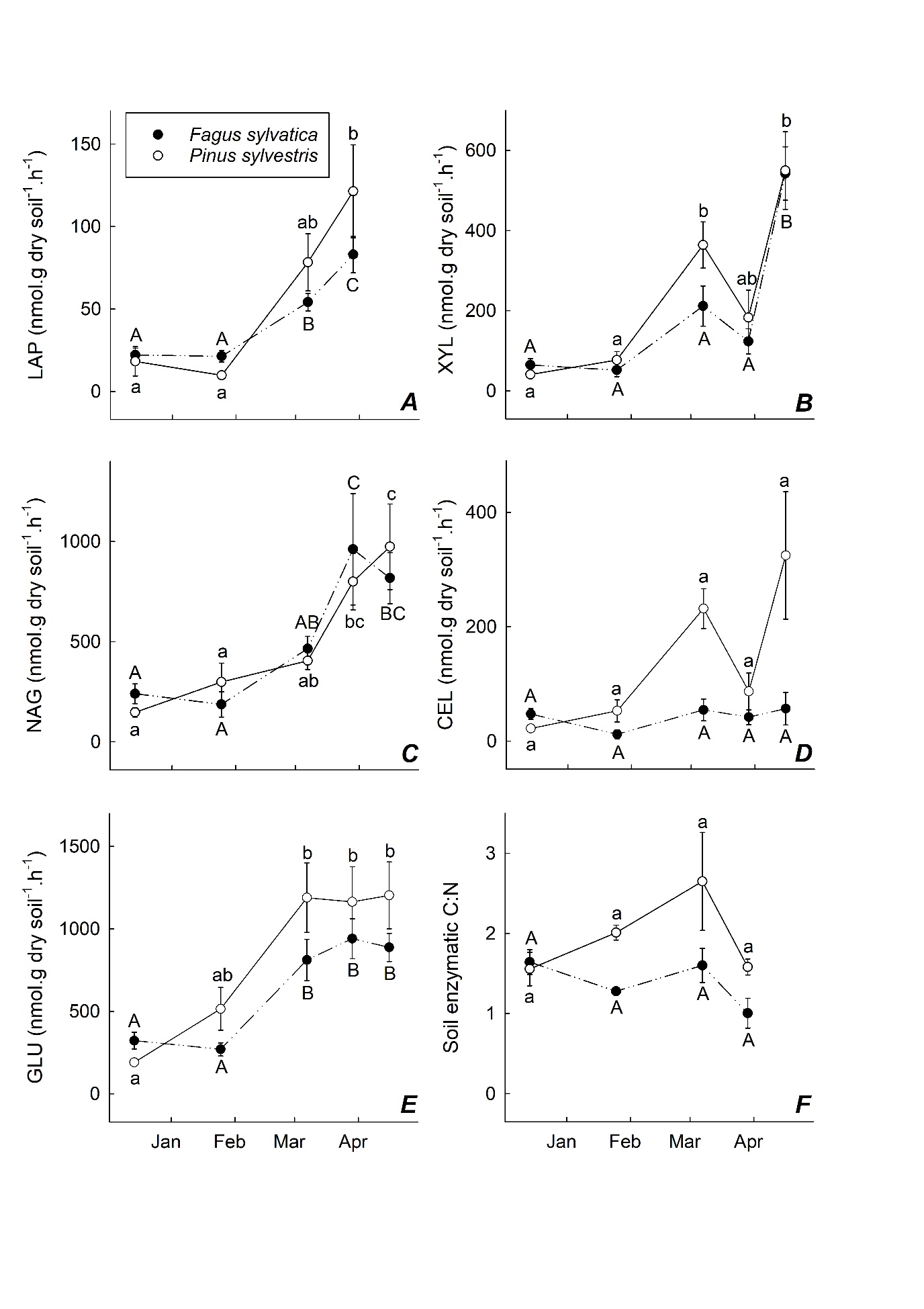


**Online Resource 3** Enzymatic activities of (A) leucine-aminopeptidase, LAP; (B) ß-xylosidase, XYL; (C) N-acetyl-glucosaminidase, NAG; (D) cellobiohydrolase, CEL; (E) ß-glucosidase, GLU; (in nmol.g dry soil^-1^.h^-1^), as well as (F) the ratio of C- to N-degrading (GLU+XYL+CEL:LAP+NAG) enzymes of the soil in the Fagus sylvatica (black dots) and Pinus sylvestris (white dots) stand from December 2015 to April 2016. LAP activity was not recorded for April 2016. Error bars denote ± 1SE (n = 5), and different uppercase (for Fagus sylvatica) and lowercase (for Pinus sylvestris) letters indicate significant differences at the p < 0.05 level
